# Experimental Study on the Aerodynamic Characteristics of a Swept-Blade Wind Turbine Under Turbulent Inflow Conditions

**DOI:** 10.3390/biomimetics11050293

**Published:** 2026-04-22

**Authors:** Junwei Yang, Chenglong Sha, Xiangjun Wang, Hua Yang

**Affiliations:** 1 Guangling College, Yangzhou University, Yangzhou 225000, China; yangjunwei@yzu.edu.cn; 2 College of Electrical, Energy and Power Engineering, Yangzhou University, Yangzhou 225127, China; x.j.wang@yzu.edu.cn; 3 College of Electrical Engineering, Yancheng Technician College Jiangsu Province, Yancheng 224000, China; 18852703430@163.com

**Keywords:** swept blade, turbulence intensity, aerodynamic characteristics, wind tunnel experiment, wind turbine

## Abstract

Avian wings enable autonomous control over flight trajectory and speed, and their swept-wing geometry inspires the application of sweep modifications to horizontal-axis wind turbine blades, an approach that is critical for improving aerodynamic performance. Hence, wind tunnel experiments were performed to evaluate the output power and wake features of a baseline straight-bladed and a swept-blade wind turbine. The experimental results demonstrate that inflow turbulence intensity (T.I.) affects the peak power coefficient of the swept-bladed turbine, with power coefficient gains being more significant when the tip speed ratio is greater than 3.0 and under yawed conditions. At a yaw angle of 20°, when the T.I. is 0.5%, 10.5%, and 19.0%, respectively, the corresponding increased values are 13.17%, 3.44%, and 4.68%. Cross-stream velocity in the near-wake region of the swept-bladed turbine is markedly higher than that for the baseline condition. The averaged T.I. in the wake velocity region of the swept-blade conditions is greater than that of the baseline condition at most measurement positions. Moreover, power spectral density (PSD) magnitudes behind the blade tip for the swept-blade configuration are higher than those of the baseline, particularly in the medium- and high-frequency domains. This work clarifies the aerodynamic characteristics of swept-blade wind turbines to varying levels of turbulent inflow.

## 1. Introduction

Confronted by the intertwined crises of dwindling energy resources and escalating environmental degradation, the development of clean and renewable wind energy has become a globally prioritized strategic emerging industry for countries worldwide. As a renewable energy source with wide distribution and enormous development potential, harnessing wind power on a massive scale is a key strategy for mitigating the impacts of global climate change [1,2]. As the core component for wind energy capture, the blade plays a decisive role in the aerodynamic efficiency of wind turbines. Therefore, further improving the wind energy conversion efficiency has emerged as an urgent and pivotal challenge in the aerodynamic optimization of wind turbine blades. After hundreds of millions of years of evolution, avians have evolved swept and curved wings with excellent aerodynamic configurations, enabling them to achieve precise control of flight attitudes. Inspired by this, researchers have carried out aerodynamic investigations on wind turbines equipped with bionic swept blades. Ding and Zhang [3] performed an optimization design of swept blades by taking the sweep onset section and the first-order derivative of the tip sweep curve as design variables, considering both annual energy production and blade root loading as integrated optimization goals. Results showed that, compared with the baseline case, the annual energy production of the swept case increased by 1.34%, accompanied by a reduction in load applied at the root of the blade, thereby lowering the operational cost of the wind turbine. Pavese et al. [4] implemented a swept design on a 10 MW wind turbine blade, which reduced the structural load of the wind turbine by decreasing the inflow angle of attack. It was found that mildly and purely shaped representations represented the optimal choice. Khalafallah et al. [5] investigated how blade sweep influences the aerodynamic behavior of wind turbines across various operating scenarios. They discovered that swept blades achieved higher power coefficients near the blade root, which increased with increasing thrust. The optimal wind turbine performance was obtained when the sweep initiation position was located at 25% of the rotor radius. Sessarego et al. [6] incorporated neural network architectures with gradient-driven algorithms to enhance the aerodynamic design of swept blades. Relative to the conventional baseline case, their optimized blades yielded an approximate 1% increase in mean power generation and an average 0.02% increase in thrust. Kaya et al. [7] adopted numerical simulations to optimize the parameters of swept blades for a 0.94 m diameter wind turbine, aiming at maximizing the power at a specified tip-speed ratio (TSR). The findings revealed a 4.28% improvement for the optimized swept-blade design when the TSR was 6.0, and the incoming flow turbulence intensity was 2.0%. Iswahyudi et al. [8] considered the sweep angle and anhedral angle in the design of three-dimensional blades to improve aerodynamic performance. Wind tunnel test results revealed that the performance improvement was attributed to the rolled-up tip vortex formed at the tip, which reduced the stall area on the inner blade by establishing a vortex-affected zone. The optimized blade achieved a maximum improvement of 37% in its output power. Iswahyudi et al. [9] also examined how variations in blade design influence the aerodynamic performance of small-scale horizontal-axis wind turbines and concluded that the swept configuration improved starting performance and reduced low-frequency noise. Pietrykowski et al. [10] developed a vertical-axis wind turbine with variable blade swept area, aiming to boost torque generation and electrical output across an extended spectrum of rotational speeds. This design adjusts the swept area of the wind turbine by changing the angles between the blade groups (ranging from 30° to 120°). Wind tunnel tests confirmed enhanced aerodynamic performance: the turbine achieved a relatively high torque coefficient of approximately 0.3 within the range of TSR (approximately 0.2–0.3) that is wider than the conventional configuration, while both the optimal TSR for maximum power and that for peak torque migrated to higher values compared to conventional configurations. Meanwhile, Veloso et al. [11] combined blade element momentum (BEM) theory with Newton’s second law to systematically analyze how blade sweep angle affects key startup characteristics, including initial torque, axial thrust, and the minimum wind velocity required for rotor initiation, in small-scale wind turbines.

In addition, Pholdee et al. [12] optimized the blade pitch angle and leading-edge curvature to maximize the torque-to-thrust coefficient ratio. Abdolahifar et al. [13] conducted three-dimensional numerical simulations to compare the aerodynamic characteristics of V-shaped wind turbines with and without twist against the baseline configuration. Within the TSR range of 0.69–1.5, the swept configuration of V-shaped blades induced inevitable spanwise flow, leading to aerodynamic performance degradation, which rendered V-shaped blade turbines less advantageous under low TSR conditions. Prakash et al. [14] proposed a novel bionic blade design drawing inspiration from the pectoral fins of the bottlenose dolphin. The swept blade altered the surface pressure distribution and promoted fluid accumulation toward the mid-span region. Their results validated a maximum torque increase of 15.7%. Li et al. [15] noted that the conventional BEM method failed to simulate the wake effect and coned rotor of swept wind turbines. Accordingly, necessary corrections were implemented for optimizing the chord length and twist angle profiles along curved blade geometries to ensure an identical spanwise circulation distribution to that of the baseline condition. The modifications eliminated differences arising from projection-based modeling, facilitating a uniform assessment of how wake dynamics influence aerodynamic loading and flow induction. In addition, Li et al. [16] proposed a streamlined engineering aerodynamic framework that integrates a near-wake vortex representation with a far-wake vortex cylinder formulation. The model was specially developed for the load calculation of swept wind turbine blades, abandoning the Prandtl tip-loss correction used in traditional BEM methods, and it can be applied to the aerodynamic study of generalized blade geometries in future research. According to the above literature review, swept blades exhibit multiple advantages: wind turbines featuring blades with an increased swept area are capable of generating greater wind energy conversion efficiency and delivering higher annual energy yields; they can also reduce blade loads to a certain extent, thus improving operational stability while maintaining favorable aerodynamic performance.

On the other hand, as rotating machinery for primary energy conversion, wind turbines operating downstream usually suffer from reduced inflow velocity and drastic variations in the angle of attack along the rotating blades due to the non-uniform natural inflow and wake effects, forcing wind turbines to operate under off-design conditions for extended periods [17]. In addition, yaw, gusts, high T.I., and complex surrounding terrain can impose unsteady loads on rotating blades, which further reduce wind energy conversion efficiency, cause output power fluctuations, and generate fatigue loads and noise. It is worth noting that vertical-axis wind turbines (VAWTs) and horizontal-axis wind turbines (HAWTs) respond differently to turbulence. The rotational plane of HAWT rotors is perpendicular to the inflow, and the blades periodically enter and exit the wind shear zone and wake region. In contrast, VAWTs exhibit more uniform stress distribution, are insensitive to wind direction, and feature relatively mild flow separation and vortex shedding, leading to lower sensitivity to T.I. Therefore, the influence of inflow T.I. should be comprehensively accounted for during the aerodynamic configuration optimization of HAWTs [18,19].

Relevant studies have confirmed the above issues. Talavera and Shu [20] conducted wind tunnel tests on an individual wind turbine as well as a pair of turbines operating under both low turbulence and turbulent flow states. Results showed strong correlations between the power coefficient and inflow T.I. in both single and array configurations. Yang et al. [21] also obtained similar findings through wind tunnel experiments. Ahmadi-Baloutaki et al. [22] generated three turbulent inflow conditions with T.I. = 5%, 7.5%, and 10% by placing grid structures inside the test section to evaluate the aerodynamic performance of a model wind turbine. Results indicated that turbulent inflow increased the output power and enhanced the self-starting performance. Using the movement of stirring blades in the 0.13 m active turbulence grids mounted at the test section entrance, Li et al. [23] examined the effects of three inflow turbulence intensities on rotor power and thrust coefficients, and the results showed that the output power was optimal at T.I. = 8.0%, superior to that at T.I. = 1.4% and 13.5%. Ahmadi and Yang [24] analyzed the spectral characteristics of rotor output power and velocity on the rotating plane in a low-turbulence environment employing numerical simulations integrated with the actuator line approach, revealing strong correlations between the two quantities over the entire frequency range. Besides the strong correlation with power characteristics, spatial correlations exist between extreme loads on wind turbines and flow field turbulence patterns. Tian et al. [25] examined how wind turbines respond under varying load conditions in an atmospheric boundary layer via wind tunnel tests, finding that inflow shear affected the time-averaged loads, while T.I. dominated the fatigue load behavior. Zhang et al. [26] observed that reducing the size of the downstream turbine markedly altered the upstream turbine’s wake configuration and enhanced wake re-energization through intensified large-scale vortex mixing.

In summary, the aerodynamic behavior of test wind turbines is strongly influenced by the T.I. of the inflow. However, the above studies on turbulent inflow were all based on straight-bladed wind turbines. At present, the understanding of the aerodynamic performance of swept-blade wind turbines under turbulent inflow remains inadequate. Meanwhile, relevant experimental studies are still rare owing to constraints imposed by the testing environment. Therefore, conclusions from straight-bladed turbines require verification for swept-blade configurations. To address these gaps, two grid-generated turbulent flow fields were designed in a wind tunnel, and an aerodynamic test platform for wind turbines was established in this study. Comparative experiments were conducted on a baseline blade and a swept-blade wind turbine under varying conditions. To provide a reference for the aerodynamic optimization of swept blades, this study analyzed the influence of inflow T.I. on the aerodynamic characteristics of the swept-bladed turbine. The paper is organized as follows. Section 2 outlines the experimental configuration, including the turbulent flow field configuration, the wind turbine model, and the blade sweep design approach. Section 3 presents a detailed analysis and discussion of the output power and wake characteristics under different T.I. conditions. Lastly, the key findings of this research are summarized in Section 4.

## 2. Experimental Apparatus and Setup

The experiment took place in the wind tunnel lab of Yangzhou University. The test section measures 3.0 m × 1.5 m × 3.0 m, with a maximum inflow velocity of 50 m/s [27]. An AC motor with a power of 185 kW and a rated speed of 600 r/min is installed in the wind tunnel power section. Total-pressure and static-pressure sensors are mounted at the entrance to the test section for real-time pressure monitoring. Based on the measured data, a feedback control system adjusts the motor speed to achieve precise regulation of the inflow velocity.

### 2.1. Turbulent Flow Field Modulation

**Figure 1:**
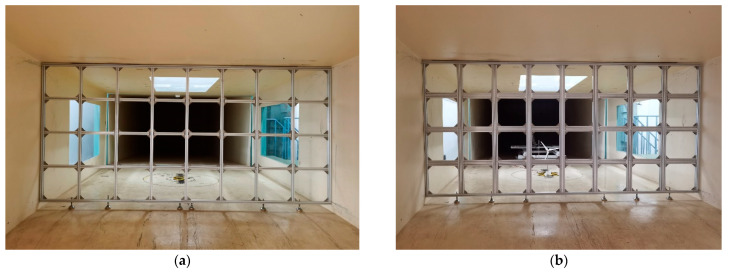
Two types of grids are used for aerodynamic comparisons of the swept-blade wind turbines. (**a**) Fine grids (Scheme 1); (**b**) Coarse grids (Scheme 2).

**Figure 2:**
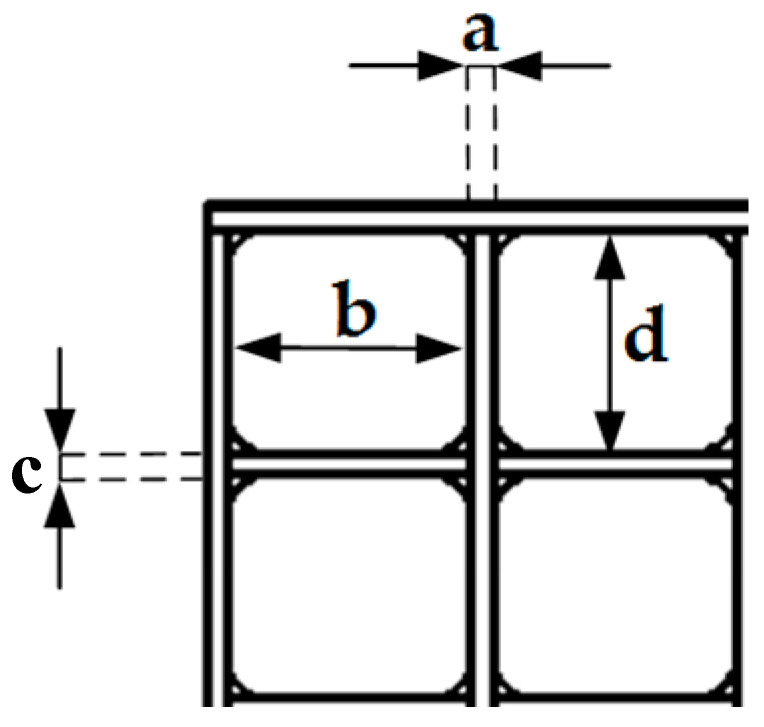
Schematic of grid parameters.

Grids are widely employed in wind tunnel experiments to generate turbulent inflows. By adjusting the size of the grids, the downstream flow structure can be modified, thereby regulating the T.I. at the test location. In the present study, two types of grids with different dimensions, as designed by our laboratory, were adopted, and their configurations are illustrated in Figure 1 and Figure 2.

The transverse bar width is denoted as a, the transverse opening width as b, the longitudinal bar width as c, and the longitudinal opening width as d. The thickness of both grids is 3.0 cm, and the detailed dimensions are listed in Table 1. In practice, rubber feet were fixed to the ground of the test section, and rubber pads were placed on the top to increase friction and ensure installation stability. A two-dimensional hot-wire probe (55P61, Dantec, Copenhagen, Denmark) was adopted for flow field measurements, with a sampling frequency of 5 kHz and a sampling time of 20 s. In the subsequent tests, the wind turbine rotor was positioned 1.6 m downstream of the grids, and the flow field was measured at the hub height of the rotor. The measurement height was approximately 48 cm above the wind tunnel floor. Data were acquired at 41 points, with an interval of 2.0 cm from the centerline to both lateral sides of the wind tunnel. It is important to highlight that the average velocity at the rotor section during data collection was 7.0 m/s, which corresponds to the target inflow speed in the following tests.

**Figure 3:**
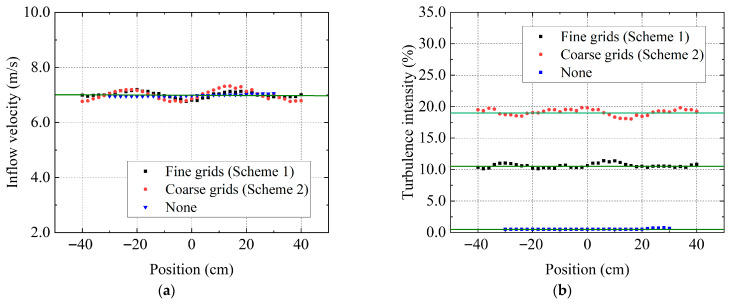
Inflow velocity and T.I. distributions without the model turbine installed. (**a**) Inflow velocity; (**b**) T.I.

Figure 3 depicts the distributions of inflow velocity and T.I. at the hub height without the model installed. The black dots denote the experimental results of scheme 1, whereas the red dots represent scheme 2. The flow field distribution without grid installation was also added as a comparative case. Due to the blockage by the grids, highly unsteady turbulent fluctuations exist in the downstream region, leading to variations at different lateral positions. It is worth noting that T.I. is calculated as the ratio of the root-mean-square (RMS) magnitude of the instantaneous velocity fluctuations to the time-averaged inflow velocity. Based on the measurements, the mean velocity of the tested flow field was 7.0 m/s. The averaged T.I. values were 10.5% for scheme 1, 19.0% for scheme 2, and 0.5% for the scheme without grid installation, which are indicated by the green lines in this figure.

**Figure 4:**
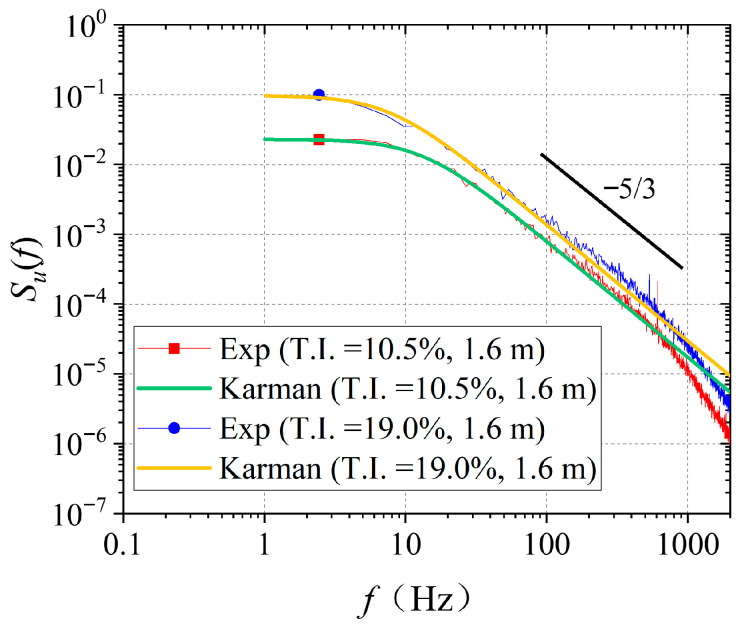
PSD of grid-generated turbulent flow fields.

Figure 4 presents the PSD of the fluctuating velocity 1.6 m downstream of the grids. The PSD distributions of the grid-generated turbulent flows agree well with the corresponding Karman spectrum [28]. The streamwise PSD function *S_u_*(*f*) is given by Equation (1):

(1) $$\frac{fS_{u}(f)}{\sigma_{u}^{2}}=\frac{4\frac{fL_{u}}{U}}{{\left[1+70.8{\left(\frac{fL_{u}}{U}\right)}^{2}\right]}^{5/6}}\;$$

In Equation (1), *S_u_*(*f*) is the PSD function of the fluctuating velocity *u* in the direction of the streamwise. 4, 70.8, and 5/6 are obtained by fitting the results from wind tunnel tests and actual measurements. It is a characteristic parameter of this empirical spectral model. *σ_u_* is the standard deviation of the streamwise fluctuating velocity *u*, and *Lu* is the streamwise integral length scale of the fluctuating velocity *u*, *U* is the mean inflow velocity, and *f* is the pulsation frequency.

It can be observed that the amplitude of *S_u_*(*f*) rises as the T.I. increases. The T.I. of scheme 1 is lower than that of scheme 2. The amplitude of *S_u_*(*f*) decreases rapidly when the frequency exceeds approximately 10 Hz, indicating that the vortices contributing most to the fluctuating energy are all below this frequency. In the inertial subrange of turbulence, the variation in PSD with frequency follows the −5/3 power law. It is used to judge whether the experimentally measured turbulence spectrum in the inertial subrange follows the energy cascade law of classical isotropic turbulence. In addition, the two cases show similar trends in the PSD. This is because grids with similar structural configurations possess an inherent similarity in the frequency-domain distribution of the energy of the generated turbulence.

### 2.2. Wind Tunnel Test Setup

**Figure 5:**
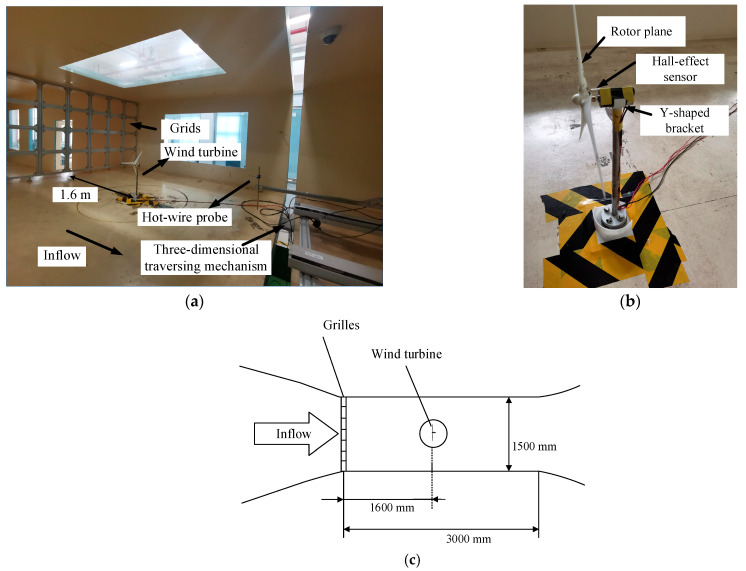
Schematic diagram and photograph of the experimental setup. (**a**) Measurement in the wind tunnel; (**b**) Model wind turbine; (**c**) Experimental arrangement.

**Figure 6:**
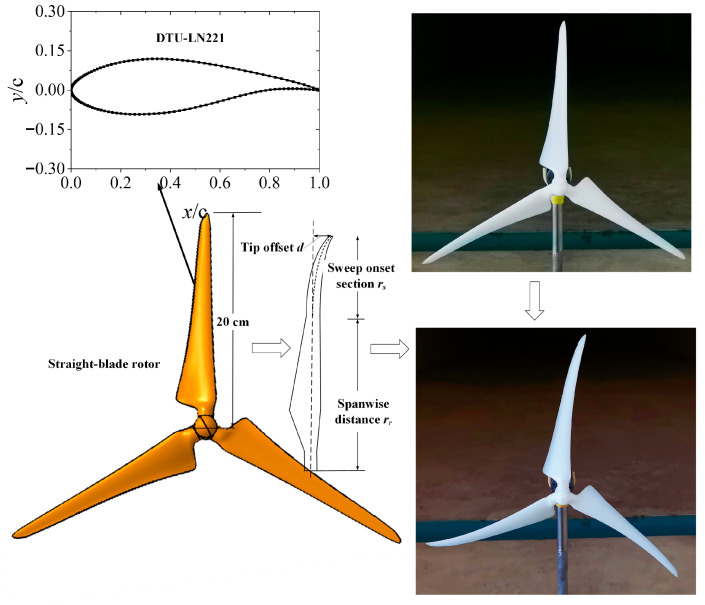
A forward-swept blade was designed in this study.

Figure 5 presents the experimental configuration, where the grid was positioned at the entrance of the test section, and two Pitot tubes were deployed 1.6 m away from the grid to record the inflow wind speed at the rotor plane of the model. The yaw angle of the model turbine could be precisely controlled by adjusting the servo motor mounted under the turntable in the test section. A miniature DC motor was fixed on the tower via a Y-shaped bracket. To avoid vibration during rotor rotation, both the tower and the base plate were made of steel. A miniature linear Hall-effect sensor of M4 size was installed downstream of the rotor for real-time rotational speed monitoring. Further details of this model wind turbine can be found in Refs. [29,30]. In this test, the rotor was fabricated using 3D resin printing. Figure 6 illustrates the baseline straight-blade rotor, which has a diameter of 0.4 m and employs the DTU-LN221 airfoil, which was originally developed by the Technical University of Denmark. The airfoil has been optimized for low-Reynolds-number conditions, effectively alleviating the insufficient spanwise velocity near the root region due to the small relative radius, as well as tip losses caused by three-dimensional effects in the tip region.

During the design of the bionic swept blades, various mathematical models have been proposed in previous studies [31,32,33] to determine the swept configuration. Most of these methods obtain the spatial offset of each blade section relative to the aerodynamic centerline through exponential equations. In this study, the blade was designed from the parameterized swept model established by Kaya et al. [34]. This model parameterizes the tip offset *d* and the sweep initiation position *r_s_*. The corresponding design formula is given by Equation (2).

(2) $$z=\frac{\left(r_{r}-r_{s}\right)\left(R\times P_{s}\right)\left(R-r_{s}\right)}{M^{\left(\frac{\left(1-P_{r}\right)\left(1-P_{rs}\right)}{P_{r}}\right)}}$$

In the equation, *z* is the offset relative to the aerodynamic centerline of the baseline case, *r_r_* is the spanwise distance, and *R* is the rotor radius. *M* represents the sweep strength, which is set to 2.0 in this study. *P*_s_, *P_rs_*, and *P_r_* are obtained from *d*/*R*, *r_s_*/*R*, and *r_r_*/*R*, respectively. In the present investigation, the sweep initiation position is set at 20% relative radius (*P_rs_* = 0.20), the tip offset is set to 10% (*P*_s_ = 0.10), meaning the blade sweep direction is consistent with the rotor’s rotational direction. In our previous uniform inflow experiments, this swept blade configuration achieved the optimal power coefficient. Therefore, the configuration is also employed when investigating the effect of T.I. [35]. It should be emphasized that the wind tunnel blockage ratio caused by the swept area under yaw-free conditions is approximately 2.79%, which is below the commonly accepted threshold of 5% for blockage correction [36]. Therefore, the wind tunnel blockage effect can be reasonably neglected. The mean inflow velocity at hub height was fixed at 7.0 m/s. This velocity was selected based on previous studies [23,37], which indicated that the influence of T.I. on the power performance becomes weaker when the inflow wind speed exceeds 8.0 m/s.

### 2.3. Measurement of Aerodynamic Power

In wind tunnel tests, a dynamic torque sensor can be installed between the model wind turbine and the motor to directly measure the output torque and thus obtain the shaft power. However, torque sensors are relatively large in size and require matching with large-scale wind turbines and generators. In this study, a miniature DC motor was adopted, which exhibits relatively low electromechanical conversion efficiency. Therefore, it is not feasible to use electrical power to characterize wind turbine performance. Owing to mechanical and electrical inefficiencies inherent in the generator, the experimentally obtained electrical power is lower than the shaft power, and the difference between them represents the power loss caused by friction torque.

**Figure 7:**
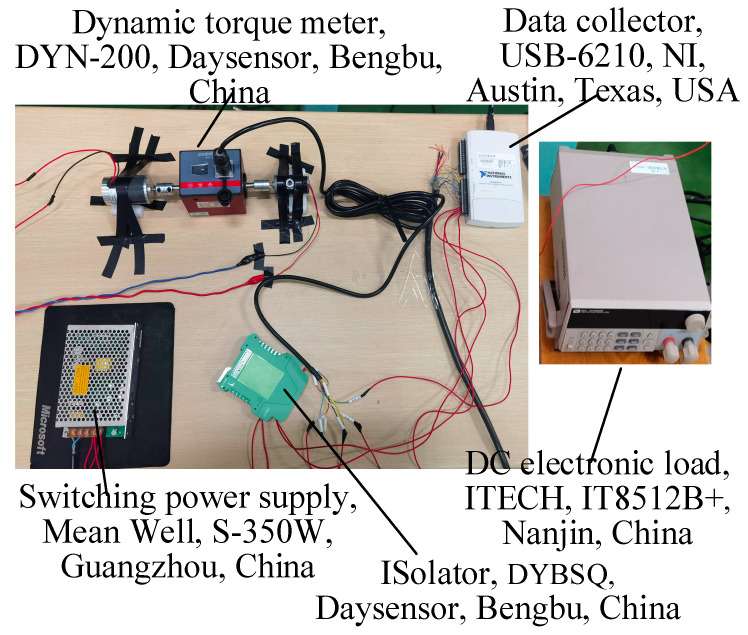
Setup for motor shaft power measurement.

To indirectly obtain the shaft power of the miniature wind turbine, a method based on fitting the shaft power as a function of armature current and rotational speed was adopted in this study [38,39]. Under steady-state operation of the generator, the armature voltage scales linearly with rotational speed, while the armature current is directly proportional to the electromagnetic torque. Additionally, the frictional torque exhibits a linear dependence on rotational speed. Consequently, shaft power can be formulated as a function of both rotational speed and armature current. In the experiments, the output voltage (slightly lower than the armature voltage), output current (i.e., armature current), and rotational speed can be directly measured. Therefore, the functional relationship among the generator’s shaft power, output current, and rotational speed is calibrated in advance, and the shaft power can be back-calculated from the measured current and rotational speed during wind tunnel tests. Since it is difficult to cover working conditions with various combinations of rotational speed and current in the wind tunnel, a dedicated calibration setup was built before the tests (as shown in Figure 7).

Two miniature DC motors were coaxially connected via a coupling and a dynamic torque transducer. One was connected to a controllable DC power supply and used as a motor, whose speed was regulated by adjusting the voltage. The other was connected to a controllable DC load and used as a generator, whose output current was controlled by adjusting the resistance. By fitting the calibrated experimental data, the functional expression among the shaft power *P*_s_, rotational speed *n*, and armature current *I* was obtained, as given in Equation (3). Additionally, our previous research has proved that the polynomial fitting shows an excellent fit with an R-squared (R^2^) value of 0.9916 and a Root Mean Square Error (RMSE) value of 0.165 W [29].

(3) $$\begin{array}{l} P_{s}=-0.1357-1.5826\times I+0.002067\times n+0.3485\times I\times I\\ +0.003805\times I\times n+0.0000002652\times n\times n \end{array}$$

In Equation (3), *P_s_* is the shaft power (W), *I* is the armature current (A), and *n* is the rotational speed (r/min). It is worth noting that a difference in order of magnitude leads to large discrepancies in the magnitudes of fitting coefficients for different terms.

In the actual power measurement, a data acquisition device (USB-6210, National Instruments, Austin, TX, USA) was used with a sampling frequency of 10 kHz and a sampling duration of 5.0 s, ensuring that each test case covered at least 50 rotor rotation cycles. The power coefficient *C_p_* is defined in Equation (4).

(4) $$C_{p}=\frac{P_{s}}{0.5\rho U^{3}\pi R^{2}}$$

In Equation (4), *C_p_* is the power coefficient, and *ρ* is the air density.

### 2.4. Wake Flow Field Measurement

As illustrated in Figure 5a, a hot-wire anemometry probe was mounted on a three-dimensional traversing mechanism via a fixed support, allowing accurate positioning within the measurement domain. It is important to note that the rotor of the model wind turbine spins in a clockwise direction when viewed from the upstream side. The hot-wire probe (55P61, Dantec, Copenhagen, Denmark) was adopted to simultaneously acquire the instantaneous streamwise and cross-stream velocities. Given the long duration of wake measurements, regular velocity calibrations using a Pitot tube were performed during the tests. Moreover, the internal temperature of the test section was kept stabilized to minimize measurement inaccuracies arising from fluctuations in ambient conditions.

**Figure 8:**
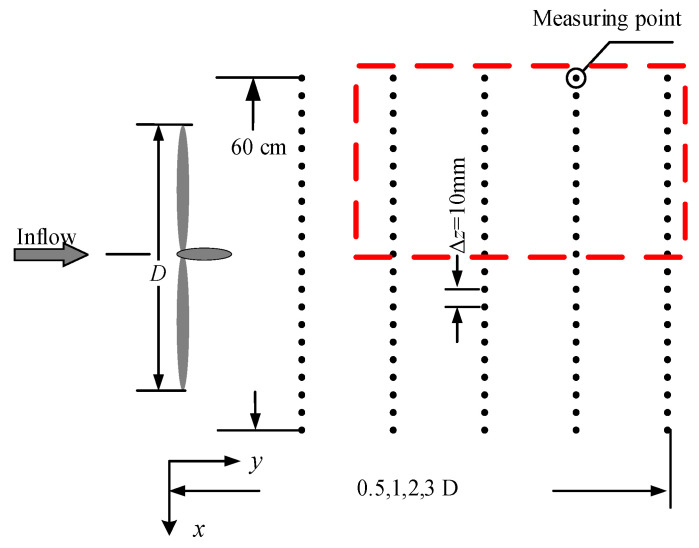
Schematic of the measuring point positions.

The measurement layout is illustrated in Figure 8. Measurement locations were positioned at the hub height across vertical planes situated at distances of 0.5, 1, 2, and 3 rotor diameters downstream from the rotor plane. Points were spaced at 1.0 cm intervals centered on the hub, extending 30.0 cm to both lateral sides, yielding a total of 61 points per cross-section. The acquisition rate of the hot-wire anemometry system was configured at 5 kHz. The arrangement for the swept cases matched exactly with the baseline cases. It is worth mentioning that previous wind-tunnel studies [40,41] have demonstrated that, when the rotor diameter is adopted as the characteristic length, flow statistics in the near-wake region become stable once the Reynolds number exceeds 9.3 × 10^4^. In other words, similar flow characteristics occur when the Reynolds number exceeds a sufficiently large threshold. In the present experiments, the corresponding value is about 1.9 × 10^5^, which is well above this threshold. Accordingly, aerodynamic characteristics in the present tests can be regarded as representative of a larger-sized wind turbine.

### 2.5. Uncertainty Analysis

The accuracy of measured data is critical to ensuring the reliability of experimental conclusions. Given the inevitable errors involved in the test procedure, an uncertainty analysis was performed. The uncertainty is divided into two categories: systematic uncertainty introduced by the measurement system itself, and random uncertainty caused by the dispersion of measured data. For systematic uncertainty, it mainly stems from the instrument accuracy of the measurement sensors. The instruments used for power measurement include the Pitot tube, Hall sensor, torque meter, and data acquisition devices. Table 2 summarizes the measurement devices along with their respective precision specifications. The inflow velocity is derived from the dynamic pressure measured by the Pitot tube, whose uncertainty is 0.1%. Meanwhile, the measurement uncertainty introduced by the corresponding data acquisition collector is 0.05%. The uncertainties introduced by the dynamic torque meter and data acquisition unit are 0.2% and 0.1%, respectively. The Hall sensor has a resolution of 0.01 Hz, the angular accuracy of the wind tunnel turntable is 1/4444.44°, and the hot-wire anemometer offers a voltage measurement precision of 1 mV, which all correspond to a measurement accuracy of 0.1%; their contributions to uncertainty are regarded as negligible. The variation in air density due to wind tunnel temperature fluctuations introduces an uncertainty of approximately 0.2%. Meanwhile, considering the manufacturing tolerance of the blades and positioning errors of the measurement points, their combined contribution to the total error is less than 1%. Based on the above analysis, the overall systematic uncertainty of the current experiments is approximated to be no greater than 3%.

For random uncertainty, due to the strong turbulent fluctuations in the inflow and flow field disruptions caused by the turbine tower and its supporting components, notable fluctuations and dispersion exist in the measured data [42,43]. To minimize the impact of such random fluctuations on the analytical results, a large number of time-series samples were recorded during the tests, and statistical averaging was applied to improve the reliability of the power and wake characteristics. In addition, to minimize random errors in wake velocity measurements, all instruments were sufficiently warmed up prior to formal testing to mitigate the influence of thermal drift on measurement accuracy.

## 3. Results and Discussion

### 3.1. Comparative Analysis of Output Power Characteristics

This section compares the variations in power coefficient with TSR for the baseline straight-bladed and swept-bladed wind turbines. The power measurement conditions are presented in Table 3.

**Figure 9:**
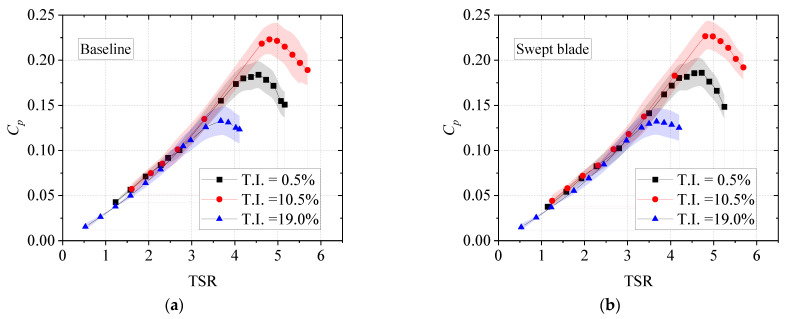
Power coefficient distributions under three turbulence intensities. (**a**) Baseline; (**b**) Swept blade.

Figure 9 shows the comparison of power coefficients under yaw-free conditions. The vertical axis in the figure represents the power coefficient, which is calculated by Equation (4). The horizontal axis is the TSR, calculated by ωR/U, where ω is the angular velocity of the wind turbine. It can be observed that the power characteristics of both turbines follow a consistent trend under the three T.I. conditions. For TSR values below 3.0, the influence of inflow T.I. on the power coefficient is relatively weak, and the power coefficients and their variation rates are nearly identical among the three inflow conditions. This is because, at a fixed mean inflow velocity, the local inflow angle variations along the rotating blade sections remain small even under high turbulence conditions. At high TSR values, however, the change in inflow T.I. significantly affects the peak power coefficient. Within the higher TSR range, the power coefficient first increases slightly with rising T.I. and then decreases sharply. For the baseline turbine, the maximum values are 0.184, 0.223, and 0.133 at T.I. = 0.5%, 10.5%, and 19.0%, respectively. The optimal power coefficient is achieved at T.I. = 10.5% over most of the TSR range. This finding aligns with the wind tunnel experimental data presented in Ref. [23]. A possible explanation is that the optimal TSR under this condition is larger than that under other conditions, yielding higher lift coefficients across each blade section and enabling the turbine to operate near its optimal condition over a broader high-TSR range. Moderate turbulence intensity (T.I. = 10.5%) improves the peak power coefficient by suppressing large-scale flow separation, promoting laminar-to-turbulent transition, enhancing airflow attachment on the suction side, and delaying detachment, thus maintaining a favorable pressure distribution and higher aerodynamic efficiency. In contrast, high T.I. introduces strong, random velocity fluctuations and large eddies, causing unsteady angle-of-attack variations, periodic blade loads, intensified 3D flow near tip and root, aggravated separation and vortex breakdown, and greater turbulent mixing losses, ultimately reducing peak power coefficient.

**Figure 10:**
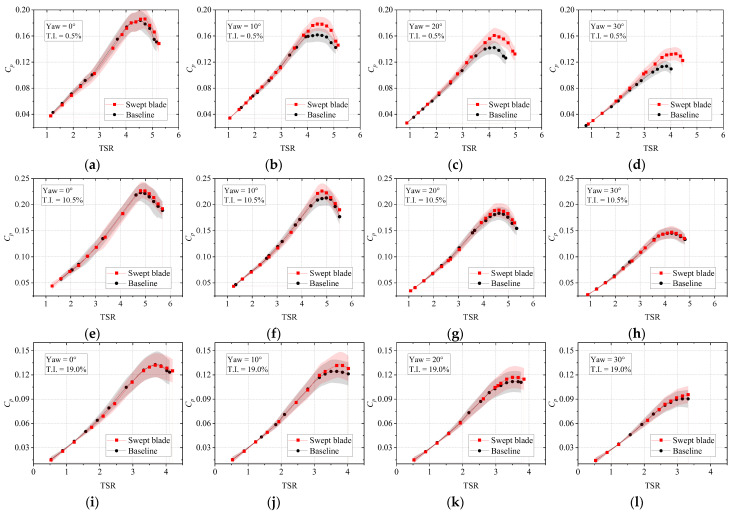
The comparisons of power coefficients at three turbulence intensities corresponding to yaw angles of 0°, 10°, 20°, and 30°. (**a**) 0°, T.I. = 0.5%; (**b**) 10°, T.I. = 0.5%; (**c**) 20°, T.I. = 0.5%; (**d**) 30°, T.I. = 0.5%; (**e**) 0°, T.I. = 10.5%; (**f**) 10°, T.I. = 10.5%; (**g**) 20°, T.I. = 10.5%; (**h**) 30°, T.I. = 10.5%; (**i**) 0°, T.I. = 19.0%; (**j**) 10°, T.I. = 19.0%; (**k**) 20°, T.I. = 19.0%; (**l**) 30°, T.I. = 19.0%.

To conduct a more comprehensive assessment of the performance improvement of the swept blade, Figure 10 compares the power coefficients under different conditions. It is evident that the difference in power coefficient between the two turbines is small at low TSR, whereas the beneficial effect of the swept blade becomes increasingly pronounced at high TSR. This might be due to the fact that, at high TSR, the baseline straight blade tends to suffer from efficiency degradation due to strengthened tip vortices and deviation of the angle of attack from its optimal range. The swept blade effectively alleviates this problem through its geometric configuration, enabling the airfoil sections to maintain a high lift-to-drag ratio. Moreover, this improvement appears to be more significant under yawed conditions. Under yaw-free conditions, the maximum enhancements in power coefficient for the three inflow conditions are 1.26%, 1.58%, and −0.54%. A previous study [35] demonstrated that, near the blade tip region, the pressure difference across the surface of a swept blade is greater than that of a straight blade. The larger effective area over which pressure is integrated further contributes to enhanced lift generation at the tip. Under turbulent inflow, the increased T.I. suppresses boundary-layer separation induced by adverse pressure gradients and intensifies momentum exchange within the boundary layer, resulting in a substantially enlarged pressure difference between the suction and pressure sides of the blade. As a result, the flow modification effects induced by blade sweep become less distinct, and its lift-enhancement role is weakened. Accordingly, the performance improvement brought by sweep is diminished at relatively high TSR conditions (i.e., TSR exceeding 3.0) and may even become negative at T.I. = 19.0% and in the yaw-free conditions. Given the stochastic nature of inflow and response delays of yaw adjustment systems, wind turbines often operate under yawed conditions in practice. Therefore, the power improvement of swept blades under yawed conditions carries important engineering significance. Whereas at a yaw angle of 20°, the corresponding values are 13.17%, 3.44%, and 4.68%. Notably, this beneficial effect is weakened under high turbulence conditions. Under yawed inflow conditions, the angle of attack undergoes relatively large periodic fluctuations. By suppressing flow separation, elevated turbulence intensity mitigates the separation risk induced by yaw, thereby weakening the performance improvement achievable with the swept blade configuration. Consequently, the magnitude of performance enhancement diminishes with the increase in T.I. Compared with other flow control methods, blade sweep as a passive flow control strategy offers the advantages of structural simplicity and strong practicality. It should be emphasized that, limited by experimental conditions, the above conclusions are only valid for low-Reynolds-number conditions at present, and further experimental validation is required for their applicability at high Reynolds numbers.

### 3.2. Verification of Wake Velocity Deficit

To guarantee the dependability of the conclusions regarding the wind turbine wake characteristics, validation of the measured data is required. In early studies, an exponential function of *y*/*D* was commonly used to fit the deficit along the centerline of a rotating turbine. The expression is given as follows [44]:

(5) $$U_{D}=A_{1}\;{\left(\frac{y}{D}\right)}^{A_{2}}$$

where *U*_D_ is the maximum dimensionless wind velocity deficit, which should be greater than zero in theory. *D* is the rotor diameter. A_1_ and A_2_ are empirical fitting parameters. However, the velocity gradient varies within different regions of the wake in wind tunnel tests, leading to non-constant fitting parameters for this semi-empirical formula. Related studies [45,46] have suggested that, under similar wind tunnel experimental setups, a Gaussian function can reasonably approximate the mean velocity distribution in the wake of a model wind turbine. For instance, Ref. [47] proposed an empirical model for the mean wake velocity distribution based on a Gaussian function. This model derives the wake velocity distribution according to the momentum theorem: when the inflow passes through the turbine, the reduction in momentum generates a thrust force, meaning that the thrust acting on the rotor equals the difference between the momentum of the flow entering and leaving the rotor plane. The empirical model was validated using tests with small-scale wind turbines and field measurements. The resulting wake velocity distribution is expressed as:

(6) $$U_{N0}=1\;-\;U_{D}e^{\frac{{\left(x_{r}-x\right)}^{2}}{2D_{r}^{2}}}$$

where *U*_N0_ is the dimensionless mean wake velocity deficit, *D*_r_ denotes the wake width at 0.5 *U*_D_, and x_r_ is the initial lateral coordinate at the wake center.

In addition, wake characteristics were experimentally investigated in a wind tunnel using a scaled-down wind turbine model, with 0.9 m diameter and 250 W rated power, as reported in Ref. [48], and the authors derived an empirical formula for the average velocity profile in the turbine wake, ignoring tower shadow interference:

(7) $$U_{N0}=1\;-\;\frac{U_{D}}{U}\;(1-\frac{r_{x}^{2}}{r_{b}^{2}}{)}^{1.5}$$

In Equation (7), r_b_ is defined as the horizontal distance measured from the center of the rotor along the x-axis to the position where the inflow is fully recovered, namely the wake radius; r_x_ is the distance along the x-axis measured perpendicular to the rotor rotational axis.

**Figure 11:**
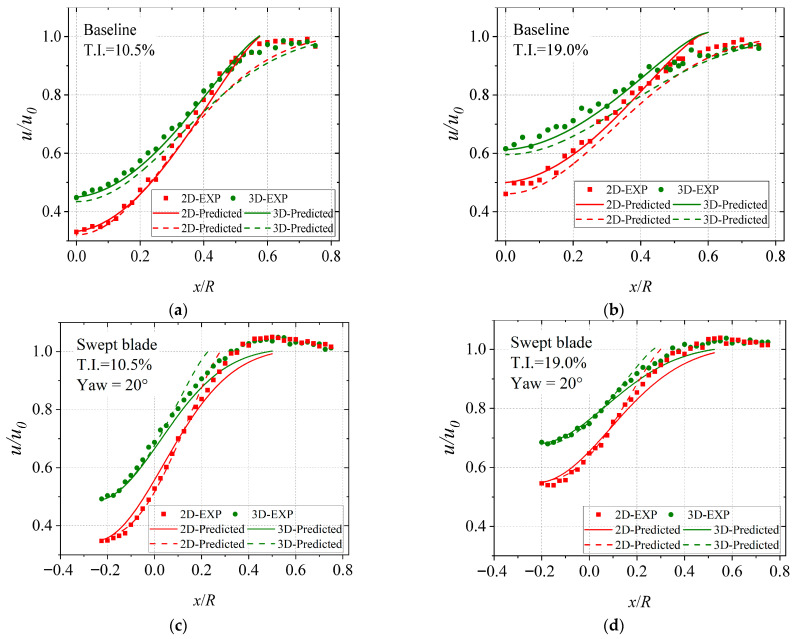
Wake mean velocity distributions and comparison with empirical models. (**a**) Baseline, T.I. = 10.5%, Yaw-free; (**b**) Baseline, T.I. = 19.0%, Yaw-free; (**c**) Swept blade, T.I. = 10.5%, Yaw = 20°; (**d**) Swept blade, T.I. = 19.0%, Yaw = 20°.

To verify the accuracy of the experimental results, using the horizontal plane located at the turbine hub height as an example, Figure 11 compares the predictions from the empirical models with the measured wake data of the baseline turbine. The experimental data are represented by scatter points, the predictions of Equation (6) by solid lines, and those of Equation (7) by dashed lines. Since the wake velocity distribution is symmetric in the horizontal direction, only the results in the positive x direction are presented. The comparison region is marked by the red dashed line in Figure 8. It can be observed that the measured mean wake velocities agree reasonably well with both empirical models. The velocity profiles exhibit self-similarity. With increasing downstream distance, the wake centerline velocity deficit decreases while the wake width increases. The wake velocity at the outer region is lower than the inflow velocity, which can be attributed to the higher power coefficient of the wind turbine at a relatively high TSR, leading to increased wake energy loss and a larger influence region, such that the wake velocity within the measurement range does not fully recover to the inflow level. Furthermore, the Reynolds number in the present tests is lower than that used for fitting the empirical formulas, corresponding to a lower power coefficient and smaller wake energy loss. This leads to a certain degree of over-prediction of the deficit by the empirical models.

### 3.3. Comparative Analysis of Wake Velocity Characteristics

**Figure 12:**
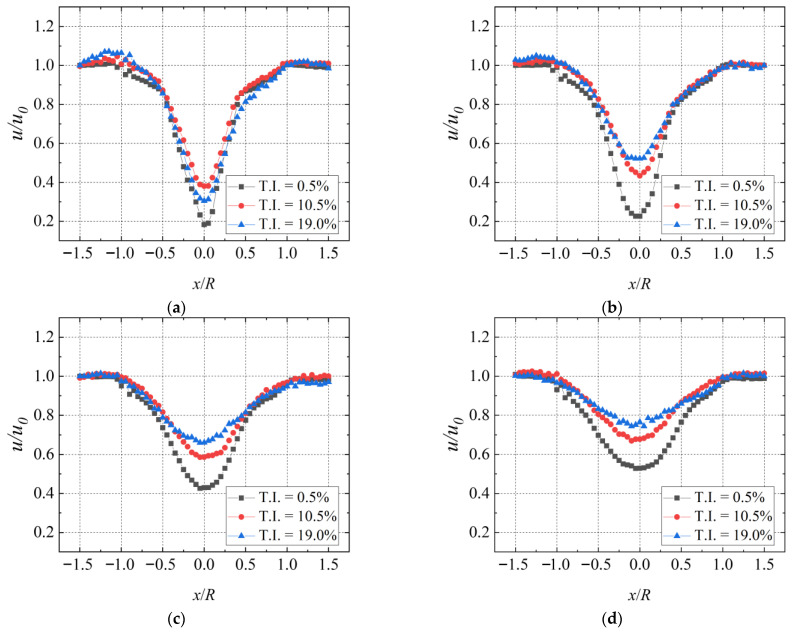
Wake streamwise velocity distribution of the baseline wind turbine at TSR = 2.0 and 0° yaw angle. (Baseline). (**a**) 0.5D; (**b**) 1D; (**c**) 2D; (**d**) 3D.

Figure 12 illustrates the mean streamwise velocity profiles within the turbine wake at a yaw angle of 0°, TSR = 2.0. It can be seen that turbulence has a considerable influence on the wake velocity recovery. At T.I. = 0.5%, the minimum time-averaged streamwise velocities at the 0.5D, 1D, 2D, and 3D planes recover to 18.3%, 22.6%, 42.6%, and 52.8% of the inflow velocity, respectively. At T.I. = 10.5%, the corresponding values are 38.1%, 43.3%, 58.6%, 67.8%; at T.I. = 19.0%, they are 30.5%, 52.2%, 66.0%, 74.5%. It is clear that the deficit is significantly reduced under turbulent conditions, indicating faster wake recovery than under low-turbulence conditions, which is consistent with observations in previous studies [49]. This behavior can be explained as follows. During turbine operation, tip vortices around the rotor, central vortices behind the nacelle, Karman vortices generated by flow around the tower, and attached vortices near the blade root interact with each other. As T.I. increases, the interaction and mixing among these vortex systems become stronger. This facilitates momentum transfer between the wake region and the adjacent flow field, thus enhancing the rate at which wake velocity recovers. That is to say, an increased inflow T.I. accelerates the wake recovery process. This phenomenon is also evident from Figure 12, which shows that turbulence has no obvious effect on the lateral shift in the wake velocity along the rotor centerline at the 0.5D and 1D positions. However, at the 2D and 3D planes, turbulence induces a slight shift in the wake center, and the shift magnitude increases with rising T.I. This is because turbulence compensates for the energy loss caused by tip vortices and core vortices within the near-wake zone. For swept blade conditions, as can be found from our previous study [35], the wake velocity difference between the baseline conditions and the swept conditions is small under different conditions. Therefore, the results of the swept blade also follow the trend shown in Figure 12, that is, the wake recovers faster with increasing T.I. The above comparisons clearly verify the accuracy of the present experimental measurements.

**Figure 13:**
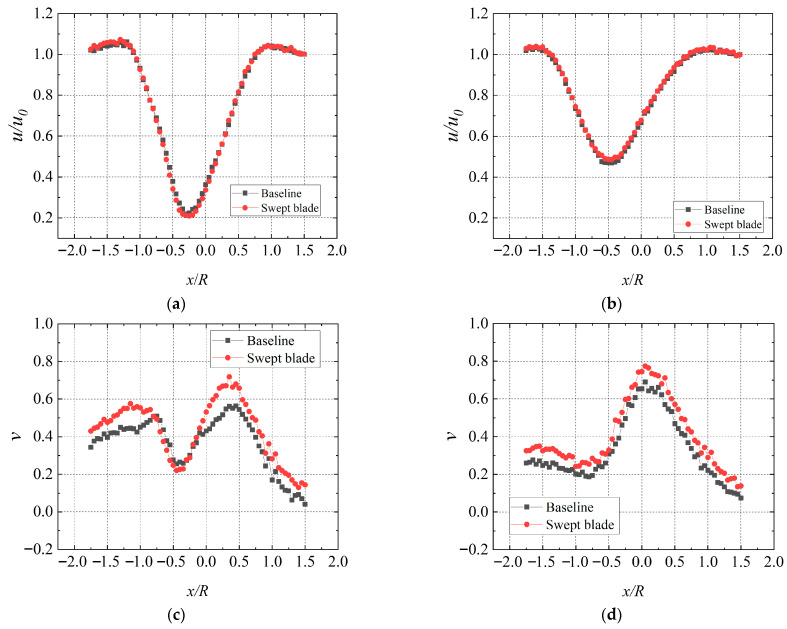
Wake velocity distribution at T.I. = 10.5%, TSR = 5.2, and 20° yaw angle. (**a**) streamwise, 1D; (**b**) streamwise, 3D; (**c**) cross-stream, 1D; (**d**) cross-stream, 3D.

To investigate the wake velocity distribution of the swept blade under different T.I. levels, wake measurement was conducted, and the relevant plan is presented in Table 4. Figure 13a,b present the time-averaged streamwise velocity distributions in the wakes of the swept-bladed and straight-bladed turbines. It can be seen that, at a relatively high TSR, the streamwise velocity distributions of the two turbines are similar in the near-wake region. At the rotor centerline on the 1D plane, the swept-bladed turbine shows a slightly larger velocity deficit and slower velocity recovery than the straight-bladed one. However, at the 3D plane, the wake velocity recovery of the swept-bladed turbine is superior to that of the baseline case. Classical momentum theory models the airflow passing through a wind turbine using an idealized control volume, a flow tube, under simplified assumptions, ignoring friction effects and the rotational velocity component in the wake. However, a rotating velocity component inevitably exists in the wake of an actual operating turbine. The rotor induces swirl in the surrounding flow field and alters the cross-stream distribution of the induced velocity, which implies kinetic energy loss in the rotating wake. Affected by the rotor, an angular velocity opposite to the rotor’s rotation direction is generated in the wake, resulting in a reverse-rotating cross-stream velocity component [50,51].

It can be observed from Figure 13c,d that T.I. and rotor rotation give rise to fluctuations in the cross-stream velocity. This is due to the evolution of tip vortices and central vortices, which lead to strong fluctuations of the time-averaged cross-stream velocity. With the diffusion of the wake and the entrainment from the surrounding flow, the cross-stream velocity gradually changes. Notably, the cross-stream velocity of the swept-bladed and baseline straight-bladed turbines shows similar trends, but the cross-stream velocity of the swept-bladed turbine is larger. A similar conclusion can be drawn at T.I. = 19.0%. The swept blade intensifies streamwise velocity loss in the near-wake region, enabling the rotor to capture more wind energy, while also increasing energy dissipation in the rotating wake.

### 3.4. Comparative Analysis of Turbulence Intensity in the Wake

**Figure 14:**
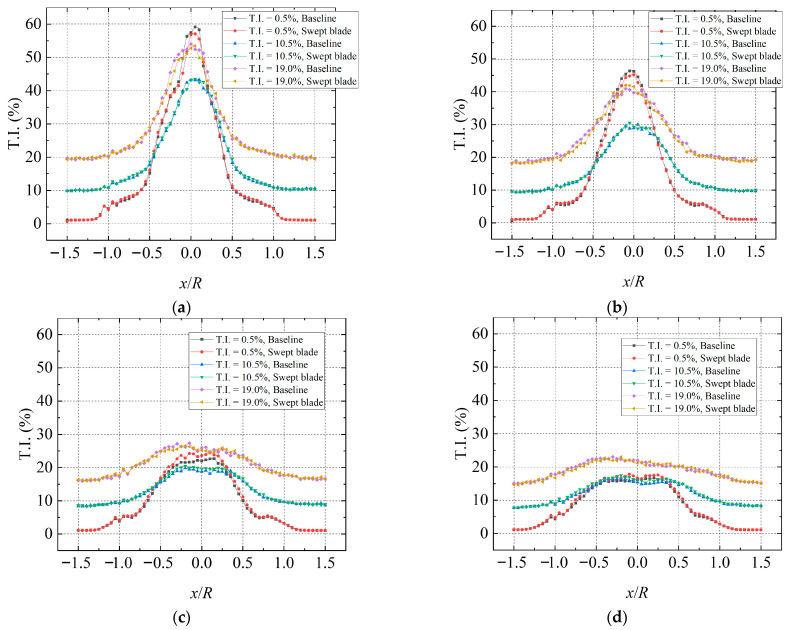
Comparison of wake streamwise T.I. at TSR = 2.0 and 0° yaw angle. (**a**) 0.5D; (**b**) 1D; (**c**) 2D; (**d**) 3D.

Figure 14 illustrates the spatial arrangement of T.I. in the turbine wake under various inflow T.I. conditions. For clear comparison, the T.I. values presented were calculated as *σ_u_*/*U*. It can be observed that a strong flow disturbance exists downstream of the rotor, which arises from the turbulent kinetic energy interaction between the turbine wake and the surrounding airflow across various vortex sizes. Observed in the horizontal plane at hub height, high T.I. mainly occurs behind the hub and spreads outward. At T.I. = 0.5%, the T.I. in the wake zone is considerably higher than the inflow T.I., which is induced by rotor rotation and becomes more pronounced in the near-wake region. Nevertheless, under high-turbulence conditions, the inflow inherently carries strong vortical structures generated by the turbulence-generating grids. Consequently, the increase in wake T.I. caused by rotor rotation is much smaller than that at T.I. = 0.5%. At the 0.5D and 1D positions, the peak T.I. in the wake reaches its maximum at inflow T.I. = 0.5%.

Moreover, the spatial influence range of wake T.I. expands with increasing inflow T.I. The swept-bladed and straight-bladed turbines show similar trends in wake T.I. distribution, but the swept-blade case exhibits a slight increase in T.I. at most measurement locations, especially in high turbulence conditions. Higher T.I. indicates that the turbine is subjected to stronger unsteady aerodynamic loads. This suggests that the aerodynamic performance improvement achieved by the forward-swept blade design is obtained at the cost of a moderate increase in structural loads. In addition, the wake T.I. characteristics of the swept-bladed turbine at high TSR were analyzed, and the increasing trend of the wake T.I. is more obvious.

**Figure 15:**
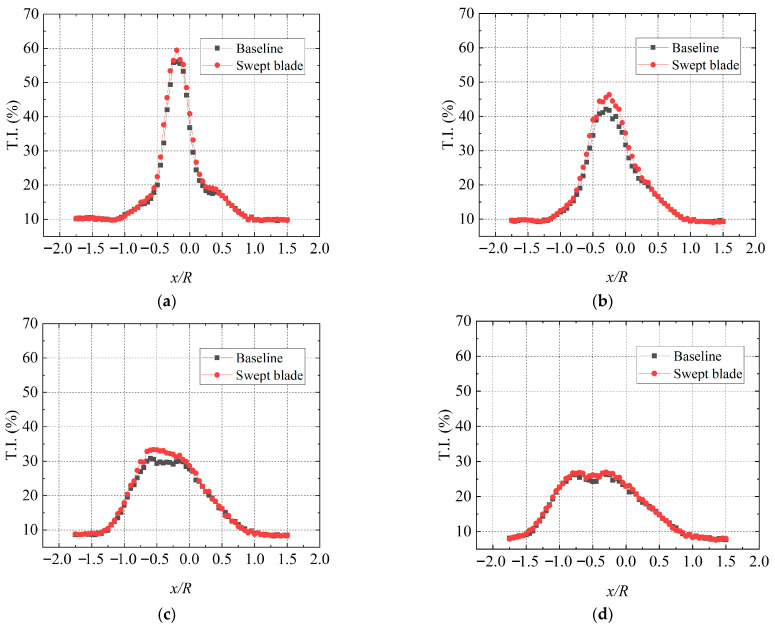
Comparison of wake streamwise T.I. at TSR = 5.2 and 20° yaw angle. (**a**) 0.5D; (**b**) 1D; (**c**) 2D; (**d**) 3D.

Figure 15 presents the T.I. distribution at different cross-sections under turbulent inflow and 20° yaw. It can be seen that the wake T.I. generally maintains a relatively high level at high TSR. At the 0.5D plane, the peak wake T.I. values reach 59.4% and 56.1% for the swept-bladed and baseline straight-bladed turbines, respectively. In the near-wake region, the two turbines exhibit similar T.I. variations, but the swept-bladed turbine shows slightly higher wake T.I., with the difference being more noticeable near the wake centerline. Compared with low TSR conditions, high TSR leads to greater wake energy loss, a larger influence region, and a significant rise in wake T.I. A similar trend is also observed at T.I. = 19.0%. Obviously, blade sweep increases the streamwise T.I. in the near-wake region, and such an increase is particularly evident at the hub centerline.

### 3.5. Comparative Analysis of Power Spectral Density in the Wake

**Figure 16:**
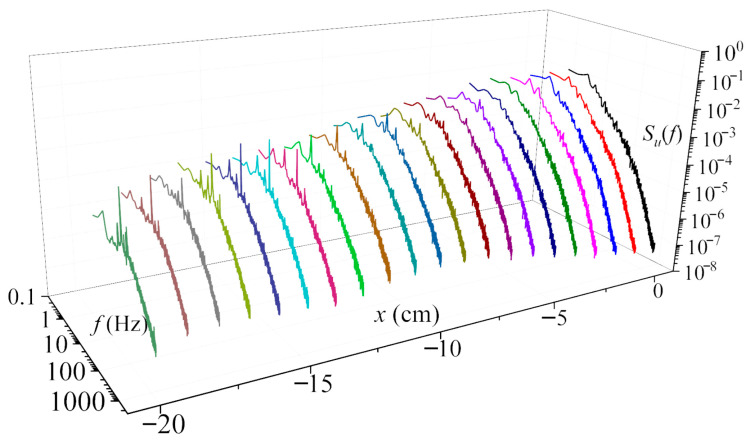
Wake velocity PSD of the baseline wind turbine at 1D (T.I. = 0.5%, TSR = 4.67, streamwise direction).

A frequency-domain analysis was performed on the measured wake velocity series to characterize the turbulent kinetic energy distribution at different wake locations. The PSD was calculated from the sampled time series using a rectangular window with a segment length of 2^10^ data points and a 50% overlap between adjacent segments to reduce spectral noise. Taking the streamwise PSD of the baseline turbine wake at 1D as an example, it can be observed from Figure 16 that, in the outer blade region (x ≤ −13 cm), the peak frequency is close to the rotational frequency of the turbine. The blade rotation frequency dominates the flow fluctuations, causing turbulent energy to concentrate near the rotational frequency and its harmonics, thereby enhancing the energy content at the fundamental and harmonic frequencies. In the inboard region, the peak frequency decreases with decreasing coordinate value, while the overall PSD amplitude gradually increases. The energy distribution in the high-frequency range follows the −5/3 power law. The increase in PSD amplitude corresponds to a higher T.I., which is consistent with the results presented in Section 3.4. It is worth noting that we also found a similar trend in the wake velocity PSD of the swept-blade conditions. The consistent experimental conditions ensure that the wake energy distribution laws and vortex structure evolution characteristics at different spanwise positions remain consistent in trend. In addition, a low-frequency peak appears in the blade inboard region, whose frequency is one order of magnitude lower than the rotational frequency. This observation is consistent with similar experiments [52], in which prominent low-frequency peaks were detected near 6 to 8 Hz. A possible explanation is that the dimensions of the generator used in the present tests are relatively large, leading to a higher nacelle-to-rotor diameter ratio than that of full-scale turbines. The blockage effect of the model generator induces vortex shedding similar to that around a bluff body. In full-scale field measurements, the nacelle is much smaller relative to the rotor, so such low-frequency fluctuations are rarely reported.

**Figure 17:**
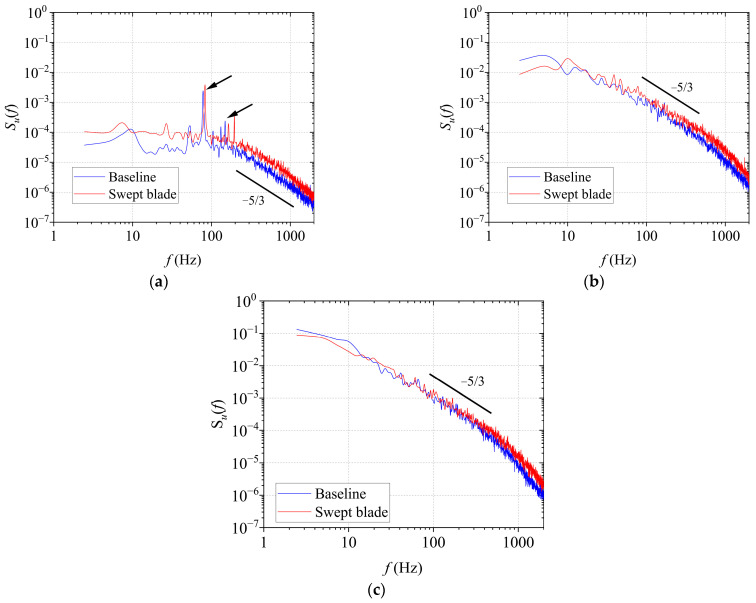
Wake velocity PSD comparison between baseline and swept-blade configurations (left tip position). (**a**) T.I. = 0.5%; (**b**) T.I. = 10.5%; (**c**) T.I. = 19.0%.

Figure 17 compares the wake velocity PSD at the left blade tip. At this location, both the baseline and swept-bladed turbines show similar trends in energy distribution, while the overall PSD amplitude is higher for the swept-blade case. Moreover, both turbines exhibit distinct peaks at the rotational frequency and its multiples. This arises from the interplay between the rotor and the surrounding flow structure, which transfers energy from the fundamental frequency to the rotating frequency and higher harmonics, with the amplitude gradually decreasing at higher frequencies. The wake velocity PSD under turbulent inflow conditions was also analyzed. A reference line with a slope of −5/3 is included to verify the transfer of kinetic energy from larger to smaller turbulent vortices within the inertial subrange. Figure 17b,c show the wake velocity PSD at the left blade tip of the baseline turbine for T.I. = 10.5% and 19.0%, respectively. As shown in the data, the peak associated with tip vortices is significantly lower than that at T.I. = 0.5% due to strong turbulent fluctuations. In addition, the PSD amplitude increases with rising T.I., indicating higher fluctuating energy in the wake. This occurs because turbulence disrupts the wake flow field and strengthens the mixing between the ambient flow and the wake. The interaction and merging of various vortical structures enhance momentum exchange across the wake boundary, thereby increasing the amplitude of fluctuating energy and promoting wake recovery. The overall PSD amplitude under the swept-blade condition remains higher than that under the baseline condition, and this difference is more pronounced in the medium-to-high frequency range. The enhanced flow instability, more active boundary-layer fluctuations, and the abundant generation and rapid evolution of small-scale vortex structures induced by blade sweep led to a more concentrated fluctuating energy associated with these high-frequency turbulent components, thereby increasing the mid-to-high frequency PSD amplitude under the swept-blade condition.

## 4. Conclusions

In this study, a swept-blade wind turbine aerodynamic test platform was established. A bionic swept design was applied to a conventional horizontal-axis wind turbine. The power and wake characteristics of the bionic swept-blade turbine were investigated at inflow turbulence intensities of 0.5%, 10.5%, and 19.0%, and the aerodynamic improvements induced by blade sweep were examined. The key findings are outlined below:

When the TSR is below 3.0, the influence of T.I. on the power coefficient is comparatively weak, and both the power coefficient and its trend are similar among the three inflow conditions. At high TSR values, however, the inflow T.I. significantly affects the peak power coefficient. The baseline turbine achieves its peak power coefficient, 0.223, under the condition of T.I. = 10.5%. While at low TSR, the power coefficient exhibits only a marginal variation between the swept-blade and baseline turbine configurations. By contrast, the improvement in power coefficient from swept blades becomes more pronounced at high TSR and under yawed conditions. Under yaw-free conditions, the maximum power coefficient enhancements observed are 1.26%, 1.58%, and −0.54%, respectively. At a 20° yaw angle, the corresponding enhancements rise to 13.17%, 3.44%, and 4.68%, respectively. It is worth noting that this beneficial effect tends to be weakened under high turbulence conditions.

Inflow T.I. has a considerable influence on the wake velocity recovery. A higher inflow T.I. leads to faster streamwise velocity recovery. For the forward-swept-blade turbine, the mean streamwise wake velocity within the near-wake zone shows a similar trend to that of the baseline turbine. However, the cross-stream velocity in the near-wake region is considerably higher. In this experiment, the maximum difference value at the 1D position was approximately 0.2 m/s. Clearly, the swept blade design increases the energy dissipation in the rotating wake. The results of wake T.I. indicate that a higher inflow T.I. corresponds to a wider influence region on the wake T.I. distribution. The wake T.I. of the forward swept-blade turbine shows a slight increase at most positions compared with the baseline case, particularly in the region close to the central axis. That is, the swept-blade design increases the streamwise T.I., and the aerodynamic performance improvement is achieved at the cost of a moderate increase in structural loads.

In addition, PSD analysis was performed to examine the turbulent kinetic energy distribution at different wake positions. At T.I. = 0.5%, the wake velocity PSD at the blade tip exhibits prominent peaks at the triple rotational frequency, the value is approximately 78 Hz in this experiment, and its harmonics for both the baseline and swept-blade turbines. Under turbulent inflow, these harmonic peaks are significantly weaker than those under low-turbulence conditions. Meanwhile, the PSD amplitude under the forward swept-blade case is higher than that of the baseline, and this feature is more evident in the medium-to-high frequency range. The results presented herein offer valuable insights to guide the aerodynamic design of swept blades.

As a promising passive flow control method, bionic blade sweep is applicable at least for small-scale horizontal-axis wind turbines. By correcting aerodynamic data for Reynolds number and three-dimensional flow effects, and validating the corrected data against field measurements, the model test could establish a reliable foundation for full-scale wind turbine design. However, this study does not consider the effects of wind shear, tower shadow, and pitch angle on the aerodynamic performance of swept blades, introducing a certain discrepancy between the present results and the actual operating conditions of full-scale turbines. Moreover, the quantitative impact of varying blade sweep configurations on turbine aerodynamic performance warrants further investigation and will be addressed in subsequent work.

## Figures and Tables

**Table 1:** Geometric parameters of the grids.

	a (mm)	b (mm)	c (mm)	d (mm)
Fine grids	30	340	30	320
Coarse grids	60	314	60	298

**Table 2:** Accuracy of instruments.

Parameter	Sensor	Accuracy	Range
Dynamic pressure	YLC-08 pitot tube Suzhou, China	±0.1%	2–70 m/s
Pressure measurement system	DTC Initium Hampton, VA, USA	±0.05%	±2500 Pa
Dynamic torque meter	DYN-200 Bengbu, China	±0.2%	±0.3 Nm
Data acquisition	NI USB-6210 Austin, TX, USA	±0.1%	±10 V
Switching power supply	MeanWell S-350W Guangzhou, China	±1.0%	Supply voltage: 12 V
DC electronic load	ITECH IT8512B Nanjing, China	±0.1%	0–10 V 0.1–100 Ω
Hot-wire anemometer	Dantec Dynamics A/S Copenhagen, Denmark	±0.1%	0–50 m/s
Hall sensor	XJERDQ M4 Wenzhou, China	±0.1%	0–10 kHz

**Table 3:** Power measurement plan.

Wind Turbine	Yaw Angle	Test Grids
Baseline, swept-blade	Yaw-free, 10°, 20°, 30°	None, scheme 1, 2

**Table 4:** Wake measurement plan.

Wind Turbine	Measurement Conditions
Baseline, Swept-blade	T.I. = 0.5%, 10.5%, 19.0%, TSR = 2.0, Yaw-free
	T.I. = 0.5%, TSR = 4.67, Yaw-free
	T.I. = 10.5%, TSR = 5.2, Yaw = 20°
	T.I. = 19.0%, TSR = 3.7, Yaw = 20°

## Data Availability

The data can be obtained upon request from the corresponding author.
